# Poly(vinyl alcohol)-Based Biofilms Plasticized with Polyols and Colored with Pigments Extracted from Tomato By-Products

**DOI:** 10.3390/polym12030532

**Published:** 2020-03-02

**Authors:** Laura Mitrea, Lavinia-Florina Călinoiu, Gheorghe-Adrian Martău, Katalin Szabo, Bernadette-Emoke Teleky, Vlad Mureșan, Alexandru-Vasile Rusu, Claudia-Terezia Socol, Dan-Cristian Vodnar

**Affiliations:** 1 Institute of Life Sciences, University of Agricultural Sciences and Veterinary Medicine, Calea Mănăştur 3-5, 400372 Cluj-Napoca, Romania; laura.mitrea@usamvcluj.ro (L.M.); lavinia.calinoiu@usamvcluj.ro (L.-F.C.); adrian.martau@usamvcluj.ro (G.-A.M.); katalin.szabo@usamvcluj.ro (K.S.); bernadette.teleky@usamvcluj.ro (B.-E.T.); 2 Faculty of Food Science and Technology, University of Agricultural Sciences and Veterinary Medicine, Calea Mănăştur 3-5, 400372 Clu-Napoca, Romania; vlad.muresan@usamvcluj.ro; 3 CENCIRA Agrofood Research and Innovation Centre, Ion Meșter 6, 400650 Cluj-Napoca, Romania; rusu_alexandru@hotmail.com (A.-V.R.); clausocol@yahoo.com (C.-T.S.)

**Keywords:** poly(vinyl alcohol), glycerol, 1,3-propanediol, 2,3-butanediol, plasticizer, tomato pigments, rheology

## Abstract

In the current work the physicochemical features of poly(vinyl alcohol) (PVOH) biofilms, enriched with eco-friendly polyols and with carotenoid-rich extracts, were investigated. The polyols, such as glycerol (Gly), 1,3-propanediol (PDO), and 2,3-butanediol (BDO) were used as plasticizers and the tomato-based pigments (TP) as coloring agents. The outcomes showed that β-carotene was the major carotenoid in the TP (1.605 mg β-carotene/100 DW), which imprinted the orange color to the biofilms. The flow behavior indicated that with the increase of shear rate the viscosity of biofilm solutions also increased until 50 s^−1^, reaching values at 37 °C of approximately 9 ± 0.5 mPa·s for PVOH, and for PVOH+TP, 14 ± 0.5 mPa·s in combination with Gly, PDO, and BDO. The weight, thickness, and density of samples increased with the addition of polyols and TP. Biofilms with TP had lower transparency values compared with control biofilms (without vegetal pigments). The presence of BDO, especially, but also of PDO and glycerol in biofilms created strong bonds within the PVOH matrix by increasing their mechanical resistance. The novelty of the present approach relies on the replacement of synthetic colorants with natural pigments derived from agro-industrial by-products, and the use of a combination of biodegradable polymers and polyols, as an integrated solution for packaging application in the bioplastic industry.

## 1. Introduction

Plastic materials are very important for modern society, and their significance in multiple applications is undeniable and well-proven [1,2,3]. Within the worldwide use of plastics, packaging represents approximately 26% of the total plastics, an indispensable element of the global economy [4,5]. Although plastics present valuable functional advantages, like low cost, versatile design, or light weight, they also have a number of negative features, such as the long-term degradation of freshwater, oceans, and soil [6,7]. Given the harmful environmental effects of the current linear economy of plastics and the future material requirements for the growing global population that is estimated to reach nine billion by 2050, a major shift towards a sustainable plastics system based on renewable materials and energy, as well as recycling is essential [8,9].

In line with the objectives of a circular economy, as well as a bioeconomy for reducing the use of fossil resources [10,11], plastics based on renewable raw materials are already being developed as carbon-neutral alternatives to fossil-based products [12,13,14]. Nevertheless, the production of plastics from renewable raw materials remains about 1% of total plastics (335 million tons in 2016). This can mainly be attributed to competition with inexpensive plastics made from fossil fuel feedstock at low costs. The supply of biomass raw materials and recycling routes poses additional challenges for biodegradable plastic materials [15,16].

There is an increasing interest in the use of biomass-derived polymers and the study of techniques for the development of biodegradable packaging, an alternative to the use of polymers from non-renewable sources for use in bioplastic packaging [17,18]. Moreover, interest in natural substances that can be used as process additives in polymers is increasing [19,20]. However, there is still only a small number of publications dedicated to the use of natural compounds in packaging [21,22].

Many bioplastics are mixtures or blends containing synthetic components, such as polymers and additives, to improve the functional properties of the finished product and to expand the range of application. If additives and pigments can also be based on renewable resources [23], we can obtain a polymer with approximately 100% weight of biodegradation compounds [24]. Could an integrated approach be the answer for a sustainable and feasible biofilm formulation in the packaging sector? The novelty of the present paper relies on the combination of biomass and biodegradable materials as an integrated solution to the non-degradable plastic materials and synthetic colorants found in the industry for packaging applications.

Among the various natural pigment compounds that can be used in biodegradable films are carotenoids, a class of compounds that are generally used as natural dyes [25]. In this regard, a highly-available, low-cost, and rich source of natural pigments is generated by the tomato processing industry. The peels, seeds, and small quantities of pulp remaining after the technological processing of tomatoes represent between 5% and 30% of the primary product [21]. According to FAOSTAT (Food and Agriculture Organization Corporate Statistical Database, 2017), the world production of tomatoes is over 180 tons. Given that tomatoes are seasonal crops and most of the final products (sauces, juice, ketchup, and canned tomatoes) are developed from August until December, the producers accumulate large quantities of by-products in a short time, which can create serious environmental pollution and it requires special management. As mentioned, important pigments have been identified and quantified in tomato processing by-products with large applicability in industries [12]. The highest amount of coloring compounds found in tomato by-products are carotenoids [24], which are a family of natural pigments synthesized by plants and some microorganisms. β-carotene is a carotenoid showing red-orange coloration due to the presence of 11 conjugated double bonds that are oil soluble. The application of byproduct-derived colorants in biodegradable polymers as natural dyes is a relatively novel developmental issue. The combination of biodegradable polymers and polyols and natural colorants allows a new generation of environmentally friendly biomass-derived packaging materials to be obtained [21].

With regard to the biodegradable polymers to be used [26], poly(vinyl alcohol) (PVOH) represents a good candidate for the backbone of biofilms because of its unique characteristics [27]. PVOH is a polymer that has a structure composed only of carbon atoms, is biodegradable, and water-soluble under aerobic and anaerobic conditions [28]. Indeed, the physical characteristics of PVOH are deeply related to its method of preparation by complete or partial hydrolysis of polyvinyl acetate. Hydroxyl groups present in PVOH can form strong hydrogen bonds between intra- and intermolecular hydroxyl groups, which result in the high affinity of PVOH to water. Therefore, PVOH is mainly used as a membrane material [29,30,31,32]. Additionally, it can be classified according to the degree of hydrolysis⁠—fully-hydrolyzed and partially-hydrolyzed, with the partially-hydrolyzed PVOH is known to be used in foods [33]. In biofilm development, PVOH can be used due to its characteristics of having good film-forming capacity, complete biodegradability, crystal modulus, and wide-ranging crystallinity [34].

**Figure 1:**
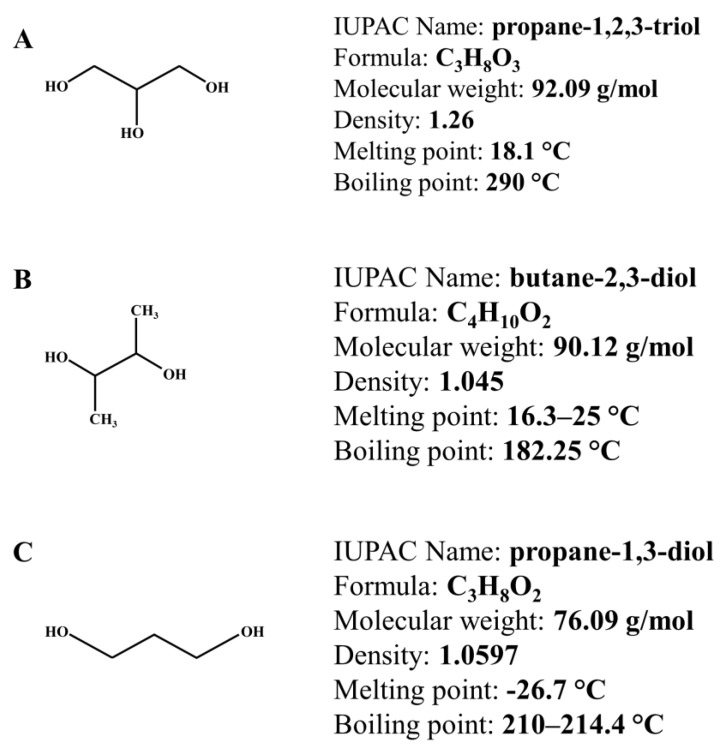
The chemical structure and properties for (**A**) (glycerol), (**B**) (2,3-butanediol), and (**C**) (1,3-propanediol).

Biodegradable PVOH-based plastics present a particular rigidity and brittleness [35], so the addition of a resistance-offering ingredient is necessary. In line with all the above, the biomass-derived polyols, such as glycerol, together with its successors, 1,3-propanediol (PDO) and 2,3-butanediol (BDO) (Figure 1), presents important plasticizer attributes in the biodegradable film’s production. It is well-known that for the production of 100 kg of biodiesel approximately 10 kg glycerol is co-produced, mainly perceived as a by-product [36]. In addition, glycerol can be produced using different processes and raw materials. For example, it can be obtained by the synthesis of propylene in several pathways, by hydrolysis of oil, or by transesterification of fatty acids/oils [37].

Propanediols are the most important value-added chemicals obtained from glycerol hydrogenolysis [38,39]. PDO shown in Figure 1C is used as an important monomer in the manufacture of polytrimethylene terephthalate (PTT) or polypropylene terephthalate, a biodegradable polyester used in textile and carpet manufacturing. Global PDO production is estimated to reach $ 621.2 million by 2021 and is growing at an annual growth rate of 10.4% [40].

Figure 1B shows BDO, also known as dimethylene glycol or 2,3-butylene-glycol, is synthesized as a product of mixed-acid fermentation [41]. The US Department of Energy classifies BDO as a chemical platform with huge potential applications in the industry. Currently, BDO is mainly produced on an industrial scale by chemical methods. However, the microbiological production of BDO offers a less expensive and more environmentally friendly alternative to traditional synthesis. This material is generated from hexoses and pentoses, mainly from the bacterial strains of the genera *Klebsiella*, *Bacillus*, *Serratia*, and *Enterobacter*, which can convert waste products (glycerol and agricultural residues) and excess biomass (wood hydrolysates) to BDO [42,43,44]. Interest in these bioprocesses has grown remarkably [45], as BDO has a large number of industrial applications, and microbial production will alleviate dependence on oil supplies for the production of chemicals [46].

The present paper, therefore, aims to combine a much debated biodegradable polymer (PVOH) with bio-based polyols (glycerol, propanediol, and butanediol) and with natural colorants (tomato-based pigments) in order to integrate the concept of the circular economy through the valorization of specific compounds obtained from biomass. The integration of plasticizers within the PVOH matrix is quite important because they improve the rheological properties of the films’ solutions, and increase the mechanical resistance of the solid films. Indeed, the PVOH is commercially available in many grades, but considering the above-mentioned aspects, we want to give low-cost alternative solutions to synthetic plasticizers, and to the synthetic colorants used for plastic materials’ dyeing, by their replacement with biomass-derived materials. The massive quantities of tomato byproducts are a valuable source of yellow/orange/red pigments that can be successfully valorized as natural colorants for biodegradable plastic materials, with specific application in packaging. Moreover, the use of natural dyeing agents in bioplastic materials makes the final product much closer to consumers. The biofilms preparation protocol was established in our previous publication [35], going further by improving the plastic properties of PVOH-based biofilms by integrating biomass-derived plasticizers in their composition. All biofilms were investigated the following aspects: carotenoid compounds found in tomato-based pigments (TP), the flow behavior of biofilm solutions, characterization of solid biofilms considering the physical appearance, FTIR analysis, UV analysis, and mechanical properties.

## 2. Materials and Methods

### 2.1. Materials

Acetic acid, acetonitrile, ethanol, and other reagents used in the experiments were of analytical grade, purchased from Sigma-Aldrich (Steinheim, Germany). PVOH with high molecular weight 98–99% hydrolyzed, 1,3-propanediol, and 2,3-butanediol was purchased from ThermoFisher (Kandel, Germany), and carotenoid standards (β-carotene) from BioMerieux (Marcy-l’Etoile, France).

### 2.2. Bioactive Compound Extraction from Tomato By-Products

#### 2.2.1. Tomato Pigments Extracted by Ultrasound

The tomato by-products derived from processing were dehydrated in a Memmert INB200 incubator oven (Memmert, Schwabach, Germany) for 48 h and finely ground. The obtained powder was extracted as previously described (in press) with ethanol 98% (1:5, *w*/*v*) assisted by ultrasound in an Elmasonic S 40 H ultrasonic bath (Elma Schmidbauer, Singen, Germany) at 37 kHz frequency and 140/340 W power, at 30 °C, for 30 min. The extracts were separated from the solid phase in a centrifuge (Eppendorf, Hamburg, Germany) at 10,000× *g* rpm and the supernatant was refined through a Millipore filter (Merck, Darmstadt, Germany) 0.45 μm. The obtained extract, further named TP, was subjected to high-performance liquid chromatography (HPLC) coupled to mass spectrometry (MS) in order to determine the total and individual carotenoid content.

#### 2.2.2. HPLC Analysis of Carotenoid Pigments

The quality and the quantity of carotenoids in the TP were analyzed by an Agilent 1200 HPLC system with a diode array detector (Agilent Technologies, Santa Clara, CA, USA). Individual carotenoids (lycopene, β-carotene, and lutein) were determined using a reversed-phase EC 250/4.6 Nucleodur 300-5 C18 ec Column (250 × 4.6 mm^2^) of 5 μm (Macherey-Nagel, Düren, Germany).

As previously described by Szabo et al. (2019), the two mobile phases were mobile phase A, consisting of a mixture of acetonitrile:water (9:1, *v*/*v*) with 0.25% trimethylamine, and mobile phase B, formed by ethyl acetate with 0.25% trimethylamine [47].

The gradient started at 0 min with 90% mobile phase A, decreased to 50% at 10 min and finished with 10% mobile phase A at 20 min, with a flow rate of 1 mL/min. The chromatograms were monitored at 450 nm, specific to carotenoids, and the peaks were identified on the basis of carotenoid standards (lycopene, β-carotene, and lutein). The amount of identified carotenoids was calculated using the calibration curve of a β-carotene standard.

### 2.3. PVOH-Based Biofilm Preparation

The biofilms were obtained by dissolving PVOH powder in distilled water at 90 °C under uninterrupted magnetic agitation for 2 h, followed by the mixture of the PVOH solution with polyols (glycerol, PDO, and BDO) and TP. The optimum ratio between PVOH, polyols, and TP concentration was established after the protocol proposed in our previous publication [35], which was 3% for PVOH, 1% for polyols, and 9% for TP. The solid biofilms were obtained by pouring 15 mL of biofilm solutions into Petri plates with 8.5 cm diameter, and letting these rest at room temperature (23 °C) for 48 h until the films were completely dried. The solid biofilms were carefully peeled off the plates and stored for one week between paper sheets at room temperature until their characterization that was performed when the solid formulations were at constant weights.

### 2.4. Flow Behavior Measurements of Biofilm Solution

Shear viscosity measurements were carried out utilizing a modular compact rheometer Anton Paar MCR 72 (Anton Paar-Physica, Graz, Austria), supplied with an air cooling concentric cylinder system (C–PTD 150/XL/AIR/18P). The used double-gap measurement system was capable of a temperature range between 5–150 °C, and each sample was allowed 5 min to equilibrate before the experiments. The measurements were carried out at three controlled temperatures 37, 30, and 21 °C, with 38 measuring points, and at a shear rate between 5–300 s^−1^. Shear rates were increased in logarithmic steps which ensured prolonged measuring point periods developing at lower shear rates, and reduced duration at higher shear rates. Apparent viscosity and shear stress were registered as a function of shear rate. To analyze the rheological data, the RheoCompass™ software was used. Each sample was measured in triplicate, and the mean values were reported further with their standard deviations (±).

### 2.5. Characterization of the Solid Biofilms

#### 2.5.1. Physical Measurements

Diameter (cm). The diameter of each biofilm was measured with a ruler. The final dimension was established as a mean value of three measurements.

Mass (g). The PVOH-based biofilms were weighted with an analytical scale (Sartorius-Acculab Atilon, Göttingen, Germany) with a precision of 0.1 mg. The final weight was the mean value of three measurements.

Thickness (mm). The thickness of biofilms was measured with a digital caliper (Lumytools LT15240, Suceava, Romania). The final thickness was the mean value of three measurements.

Density (g/cm^3^). The solid films’ density was determined by reporting the weight of the samples to their surface and thickness, according to the following equation:

*Density* (g/cm^3^) = *w*/*a*∗*t* (1)

where *w* is the weight of biofilm (g), *a* is the area of biofilm (cm^2^), and *t* is the thickness of biofilm (cm).

Moisture content. The moisture content of the solid biofilms was determined by measuring the biofilms’ weight before and after drying in the oven at 110 °C until constant values were obtained [48,49]. Samples were analyzed in triplicate. The equilibrium moisture content (%) was determined according to the following equation:

*Moisture content (%)* = (*m*_0_ − *m*)/*m*_0_∗100 (2)

where *m*_0_ is the initial weight of biofilm (g), and *m* is the final weight of biofilm (g).

#### 2.5.2. Biofilms FTIR Analysis

Fourier-transform infrared spectrometry was used for the identification of potential functional groups within the new PVOH-based formulations. For this investigation was used a Shimadzu IR apparatus (model: Prestige-21, Shimadzu Corporation, Kyoto, Japan) fitted with an ATR (attenuated total reflectance) mode with a single reflection (PIKE Technologies, Fitchburg, MA, USA) equipped with the press.

The solid PVOH-based biofilms were placed onto the horizontal accessory and analyzed against the air as background. The absorption spectra were recorded on the wavelength range of 600–4000 cm^−1^, 4 cm^−1^ resolution, and 64 scans for spectrum. The obtained results were processed using the IRsolution software programs overview (Shimadzu) and OriginR 7SR1 Software (OriginLab Corporation, Northampton, UK).

#### 2.5.3. Biofilms UV–Vis Analysis

Each type of film was cut into strips (a rectangular piece) and placed inside of a UV–Vis spectrophotometer cuvette using air as the reference sample. The measurements were done at wavelengths between 200 and 800 nm. The results have been expressed as absorbance at 600 nm. The measurements were done in triplicate and the average of three spectra was calculated. The transparency at 600 nm (T600) was calculated with the following equation [50].

*T600* (nm/mm) = *A600*/*b* (3)

where *A600* is the absorbance at 600 nm and *b* is the film thickness (mm).

#### 2.5.4. Biofilms Mechanical Properties

The mechanical properties in terms of hardness (hardness, deformation at hardness, and hardness work) were determined for each specimen by using a Brookfield CT3 texture analyzer (Brookfield Engineering Laboratories, Inc., Middleborough, MA, USA) at room temperature. The biofilm samples were cut into square pieces (3.5 × 3.5 cm^2^), clamped with grips and placed into the apparatus. For this analysis the compression test using a 2-mm diameter rod probe (TA39) at a target distance of 5.0 mm and at a test speed of 0.5 mm/s was applied. The measurements were performed in triplicate and the mean values were reported with standard deviations.

### 2.6. Statistical Analysis

The analysis was done in triplicate and the results were expressed as the mean ± standard deviation (SD). The significant differences between the transparency values of the biofilms were estimated with one-way ANOVA–Tukey’s multiple comparison test (*p* = 0.05) (GraphPad Prism Version 8.0.1, GraphPad Software, Inc., San Diego, CA, USA).

## 3. Results and Discussion

### 3.1. Characterization of Carotenoid Pigments Extracted from Tomato By-Products

Data regarding retention time, wavelength along the absorption spectrum and the quantity of each identified carotenoid compound expressed as mg β-carotene/100 DW are presented in Table 1.

The results show β-carotene, which is responsible for the orange color, was present in the highest amounts in the tomato by-product extract (1.605 mg β-carotene/100 DW), while lutein (1.498 mg β-carotene/100 DW) confers the yellow pigmentation of the biofilms. Considering the high necessity of replacing the synthetic colorants used in the packaging industry with a feasible and sustainable switch into biodegradable packaging, the TP represents a rich source of natural pigments.

### 3.2. Flow Behavior of the PVOH-Based Biofilm Solutions

The unsatisfactory thermal properties of PVOH, due to the close proximity of decomposition and melting temperatures can be enhanced with the addition of glycerol, PDO, and BDO [51,52]. Due to PVOH consisting of hydroxyl groups the bonding had a thorough effect on the mechanical and rheological features of the formed polymers. PDO and BDO are solvents with low viscosity that are able to react with the –OH groups from the PVOH [53].

**Figure 2:**
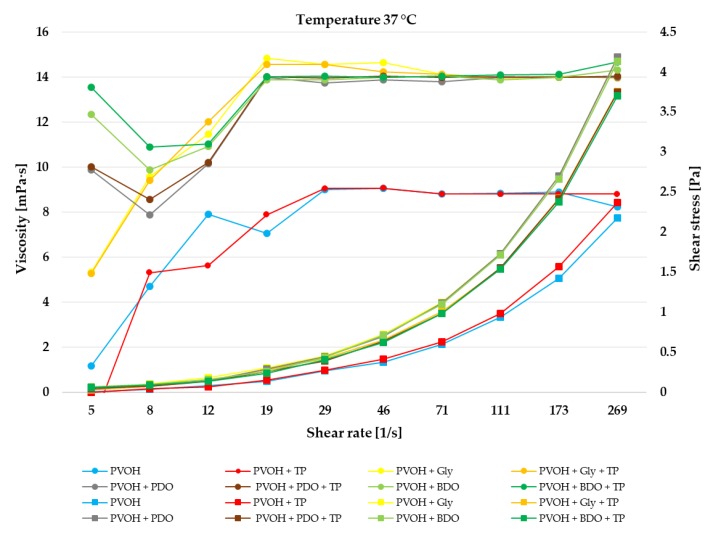
Shear viscosity and shear rate of biofilms at 37 °C temperature. The reported mean values with standard deviation (±) are available in Supplementary Material 1.

**Figure 3:**
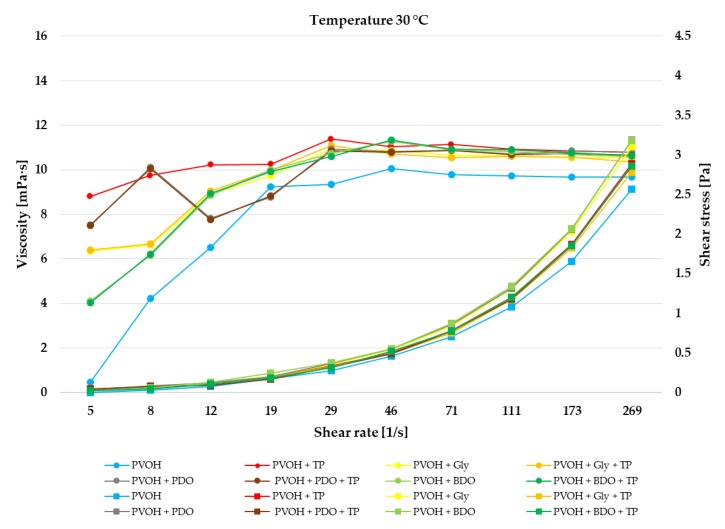
Shear viscosity and shear rate of biofilms at 30 °C temperature. The reported mean values with standard deviation (±) are available in Supplementary Material 2.

**Figure 4:**
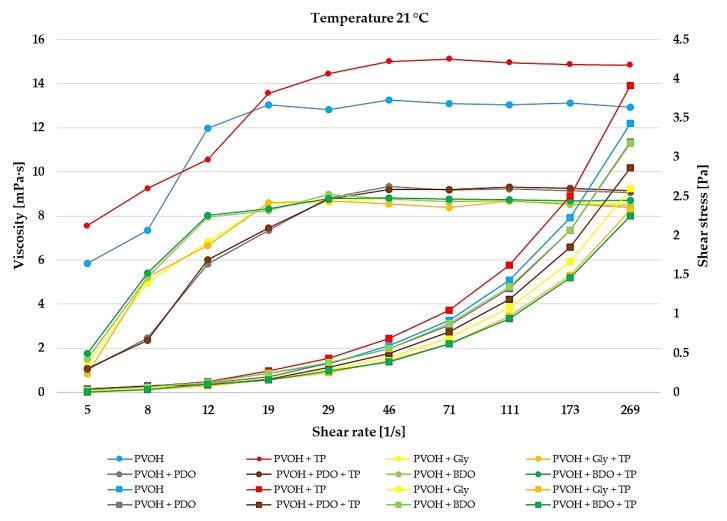
Shear viscosity and shear rate of biofilms at 21 °C temperature. The reported mean values with standard deviation (±) are available in Supplementary Material 3.

The impact of the reaction temperature on the viscosity of PVOH, PVOH+TP, PVOH+Gly, PVOH+Gly+TP, PVOH+PDO, PVOH+PDO+TP, PVOH+BDO, and PVOH+BDO+TP solutions is presented in Figure 2, Figure 3 and Figure 4. During storage, a broad range of temperatures can be found which is very important in the rheological property characterization [54].

The viscosity decreased for the PVOH, and PVOH+TP solutions with the increase of temperature from 21 to 37 °C, while for the PVOH+Gly, PVOH+Gly+TP, PVOH+PDO, PVOH+PDO+TP, PVOH+BDO, and PVOH+BDO+TP solutions the viscosity increased with the increase of temperature. According to Sukhlaaied et al. (2016), this effect is mainly a result of initiator decomposition, which generates several free radicals or active sites on each biopolymer (Gly, PDO, and BDO) backbones [55]. As a result, with the increase in temperature, the number of short chains increased accordingly. With the increase of shear rate the viscosity also increased till 50 s^−1^, reaching values at 37 °C of approximately 9 ± 0.5 mPa·s for PVOH, and PVOH+TP, and 14 ± 0.5 mPa·s in combination with Gly, PDO, and BDO. After the shear rate reached 50 s^−1^, at every temperature the viscosity remained constant.

The impact of reaction temperature on the rheological behavior is presented in Figure 2, Figure 3 and Figure 4. Additionally, in the Supplementary Materials 1–3, the shear viscosity and shear stress of biofilms at each temperature together with their standard deviation. According to these results, it can be seen that every biofilm solution presented a shear-thickening (dilatant) behavior. Dilatant fluids have a higher shear viscosity at higher shear rates, and supposedly, after a while at higher shear rates the molecular chains progressively align which had the effect of constant viscosity. The incorporation of Gly, PDO, and BDO in the biofilms had better thermoplastic processability than the simple biofilms, which had only PVOH or PVOH+TP. Workability and rigidity issues can be solved with the addition of plasticizers like glycerol which enhances the ductility and flexibility of biofilms [56,57], while PDO and BDO are able to improve the crystallinity and the thermal characteristics of biofilms [58].

### 3.3. Solid Biofilm Characterization

#### 3.3.1. Physical Appearance and Measurements

**Figure 5:**
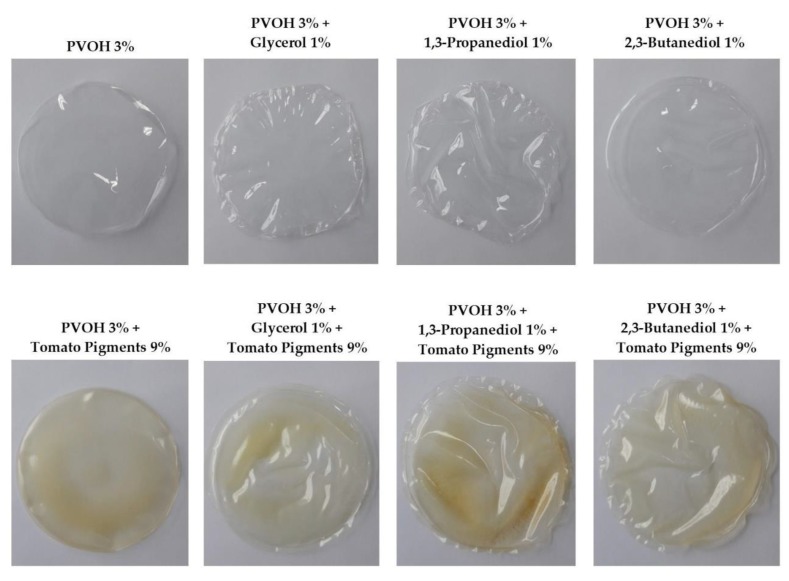
PVOH-based biofilms plasticized with polyols and colored with tomato-based pigments.

In the present study, eight types of solid biofilm were obtained (see Figure 5). The solidified PVOH-based biofilms appeared as flexible as the plastic materials that are commercially available for packaging, like the plastic materials based on polyethylene or polypropylene. The obtained biofilms were malleable and resisted easily to physical modeling. Compared with the results that we obtained previously [35] for the PVOH-based biofilms improved with chitosan or itaconic acid, the biofilms from the present study were smoother and much more flexible.

In the present experiment, the biofilm solutions cast in Petri plates thoroughly coated the surfaces, while after the water evaporated the solid biofilms were completely homogenous with no air bubbles trapped within the matrix. In addition, except for the control biofilms (PVOH and PVOH+TP) which were mechanically detached and presented a flat look, the samples containing polyols had auto-detached from the Petri plates after 48 h and showed a wavy aspect. Considering their appearance, the biofilms that contained PVOH and polyols were wholly transparent with no color shades, while the samples consisting of PVOH, polyols, and pigments from tomatoes had a yellowish color with different overtones. Since the biofilm solutions were perfectly homogenized after the magnetic stirring, the inhomogeneity in the color distribution during solidification might be due to Brownian motion and colloidal interactions [59] between pigments and other compounds from the matrix such as PVOH, glycerol, PDO, and BDO. In terms of the natural colorants as with the pigments extracted from tomato by-products (peels and seeds), they did not influence the biofilms’ malleability but confirmed their aesthetic impact in coloring the biofilm matrices regardless of the presence of polyols. For all tested biofilms, the samples that contained glycerol were the most malleable and the softest to touch, followed by samples containing PDO and BDO.

Color is an important parameter in the design of the external packaging of certain products, and it becomes a crucial factor when it is about the color of packaging for food products. In this context, alternative and sustainable solutions to synthetic colorants can be considered especially by using natural extracts based on vegetable by-products [60,61]. As the literature points out, pigments of vegetable origin (extracted from tomato peels, green tea, mint, olive leaves, pomegranate peels, sea buckthorn, etc.) abound in multiple colorful organic compounds which are sources of natural colorants and also have bioactive potential for the health of living organisms [27,60,62,63,64]. For example, Yong et al. reported that intense blue and black color was obtained from purple and black rice, and was impregnated into chitosan-based films [65]. Their results showed that the dark pigments extracted from rice imprinted a strong color and a higher resistance to the obtained films even at a low concentration of 1% (wt). The dark colors of the rice extracts were due to the high anthocyanin content of purple and dark rice [65].

**Figure 6:**
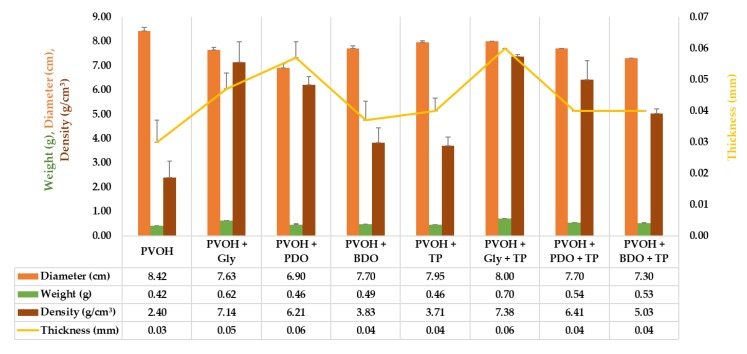
Physical measurements of solid biofilms based on PVOH. Reported data represents the average value of three replicates with their standard deviations (SD).

The physical measurements of diameter, weight, thickness, and density were measured for every individual specimen, and the results are presented in Figure 6, as the mean values of the three replicates. The maximum diameter was recorded for the control sample (PVOH) which was 8.423 ± 0.14 cm. The addition of polyols considerably reduced the diameters of biofilms to under 8 cm: 7.633 ± 0.11 cm for PVOH+Gly, 6.90 ± 0.17 cm for PVOH+PDO, and 7.70 ± 0.10 cm for PVOH+BDO. While the addition of tomato extracts extended the diameter of biofilms containing polyols: 8.00 ± 0.00 cm for PVOH+Gly+TP, 7.70 ± 0.00 cm for PVOH+PDO+TP, and 7.30 ± 0.00 cm for PVOH+BDO+TP. In the control sample based on PVOH and vegetal extract (PVOH+TP) the diameter had reduced to 7.95 ± 0.07 cm.

As was expected, weight, thickness, and density of samples increased with the addition of polyols and TP. The weight of the biofilms varied from 0.42 ± 0.00 g for the PVOH sample to 0.70 ± 0.00 g for the PVOH+Gly+TP sample. Biofilm density varied from 2.40 ± 0.06 cm^3^ (PVOH sample) to 7.38 ± 0.07 cm^3^ (PVOH+Gly+TP sample). Thickness, which is a crucial parameter correlated with important physical qualities of biofilm such as transparency/opacity, flexibility, and firmness as the literature suggests, recorded a low value for the PVOH sample (0.03 ± 0.00 mm) and a high value for the PVOH+Gly+TP sample (0.06 ± 0.00 mm) [65].

The moisture content (%) of the PVOH-based formulations ranged between 3.73% and 26.91% (Table 2). The highest moisture content (26.91%) was recorded for PVOH+Gly compared with the rest of the samples. The elevated value for moisture content might have been associated with the hygroscopicity of the polyols (glycerol, mainly) [49] that directly absorb water. Apparently, the formulations that contained TP recorded lower values of moisture content (%), a fact that could be attributed to the hydrophobicity of carotenoids [17,47], and furthermore, which could be connected with the matrix stability.

PVOH is an eco-friendly compound with ideal film-forming properties [66,67], which in mixture with diols like PDO and BDO, or triols like glycerol achieves good behavioral properties in terms of flexibility and elasticity, and maintains favorable external features like transparency and soft texture. Remarkable results were reported in the literature in the case of polyols used as plasticizers [57]. For example, Boonsuk et al. (2020) obtained biodegradable PVOH/chitosan-based films with superior mechanical properties when they added glycerol as a plasticizer [57]. PDO was intensively used as a plasticizer material in the composition of biodegradable films, fibers, and plastics of Sorona family (PTT-based plastic materials) [68]. A study conducted by González et al. in 2017 used PDO and D-isosorbide as a green plasticizer for starch-based biofilms, and evaluated the plasticizer effect on aging (two weeks) [69]. It appeared that biofilms containing PDO presented significant evolution in terms of mechanical properties and contact angle values during storage [69].

Among the tested polyols within this work, BDO is less studied as a plasticizer in PVOH-based biofilms, even though it is largely used as an intermediate compound in hard rubber product manufacturing, and also in the pharmaceutical industry as a mediator [70].

#### 3.3.2. FTIR Analysis

**Figure 7:**
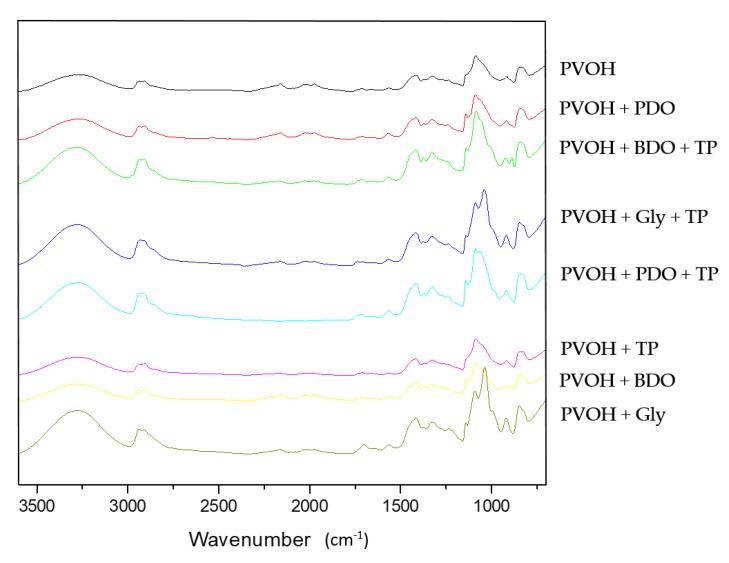
The FTIR results of PVOH-based biofilms plasticized with polyols and colored with tomato-based pigments.

Fourier-transform infrared spectroscopy is a very utilized method for the detection of chemical constitutions and possible interactions between polymer compositions [71,72]. The FTIR results obtained for the present work are illustrated in Figure 7. All samples recorded a large spectrum of different heights around 3300 cm^−1^ (PVOH/PVOH+TP: 3277 cm^−1^,PVOH+Gly/PVOH+Gly+TP: 3280 cm^−1,^ PVOH+PDO: 3275 cm^−1^, PVOH+PDO+TP: 3278 cm^−1^, and PVOH+BDO/PVOH+BDO+TP: 3278 cm^−1^), results that are associated with the presence of inter- and intramolecular hydrogen-bonded –OH groups and C–H stretching, respectively [66,73,74]. All samples recorded two small signals between 2800 and 3000 cm^−1^ (2906 and 2939 cm^−1^ for PVOH/PVOH+PDO/PVOH+BDO, 2908 and 2395 cm^−1^ for PVOH+PDO+TP/PVOH+BDO+TP, 2910 and 2937 cm^−1^ for PVOH+Gly, and 2915 and 2933 cm^−1^ for PVOH+Gly+TP) that are attributed to C–H stretches [75]. The peaks situated between 1500 and 1700 cm^−1^ (1564 and 1656 cm^−1^ for PVOH/PVOH+BDO, 1566 and 1654 cm^−1^ for PVOH+PDO, 1564 and 1641 cm^−1^ for PVOH+Gly, 1566 and 1660 cm^−1^ for PVOH+TP, 1568 cm^−1^ for PVOH+PDO+TP, 1568 and 1662 cm^−1^ for PVOH+BDO+TP, and 1568 and 1654 cm^−1^ for PVOH+Gly+TP) are related to the presence of C=C vibration, as the literature points out [76,77]. High peaks were identified around 1000 cm^−1^ especially for the samples that contain plasticizers (1087 and 1141 cm^−1^ for PVOH+PDO, 1087 and 1139 cm^−1^ for PVOH+PDO+TP, 1085 and 1141 cm^−1^ for PVOH+BDO, and 1083 and 1139 cm^−1^ for PVOH+BDO+TP. In addition, 1037 and 1141 cm^−1^ for PVOH+Gly, 1039 and 1139 cm^−1^ for PVOH+Gly+TP), a fact that indicates the presence of C–O bonds specific to diols and triols [66,74,76]. The presence in TP of compounds such as β-carotene and lutein can be associated with the small peaks observed around 1500 cm^−1^ for the samples supplemented with TP (1566 cm^−1^ for PVOH+TP and 1568 cm^−1^ for PVOH+PDO+TP/PVOH+BDO+TP/PVOH+Gly+TP) peaks that indicate C=C bonds [77].

#### 3.3.3. UV–Vis Analysis

**Figure 8:**
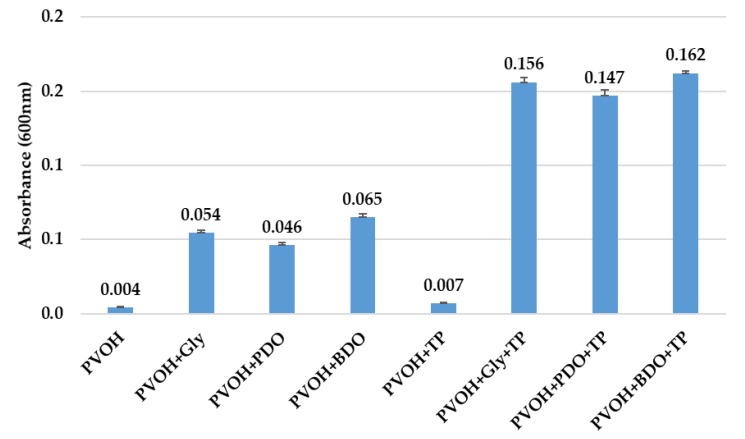
The UV–Vis results of PVOH-based biofilms plasticized with polyols and colored with tomato-based pigments. Reported data represents the average value of three replicates with their standard deviations (SD).

Film transparency has high relevance for the packaging industry, particularly if the polymer film must protect the food from the effects of light, especially UV radiation. Therefore, a high clarity is not desired, considering the need for a low transmittance to light in the UV spectrum for preventing the food-component oxidation. In Figure 8 shows the absorbance at 600 nm of PVOH-based biofilms. The biofilms’ light transmission was measured at wavelengths between 200 and 800 nm and the spectra were recorded. Biofilms with TP had lower transparency values compared with control biofilms (without vegetal pigments) suggesting that polyol-based PVOH biofilms with TP have important protection properties against ultraviolet light. These results are in agreement with the previous findings of Kanatt et al. (2012) and of Gómez-Guillén et al. (2007) who reported that there was an improvement in light barrier properties when a natural extract was added to biopolymer-based PVOH biofilms [78,79].

The effects of TP on biofilms transparency are shown in Table 3. Biofilm with only PVOH content was more transparent (lower value) than others. The incorporation of TP reduced transparency. Siripatrawan and Harte (2010) found that chitosan-based biofilms with the addition of green tea extract were less transparent than without the extract [80]. Moreover, the incorporation of phayom wood extract into the hydroxypropyl methylcellulose-based biofilms reduced their transparency [81].

#### 3.3.4. Mechanical Properties

**Figure 9:**
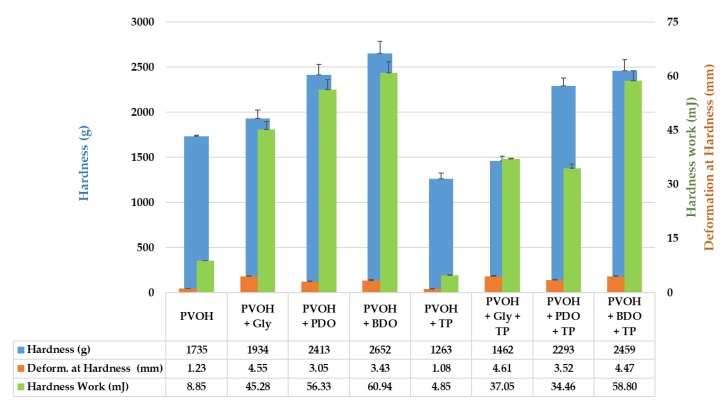
Mechanical properties of PVOH-based biofilms. Reported data represents the average value of three replicates with their standard deviations (SD).

The results for biofilm hardness are shown in Figure 9. Considering the hardness parameter, which represents the maximum load value of the compression cycle to attain the maximum deformation [82], the highest values for the biofilms containing BDO and PDO as plasticizers with and without the presence of coloring agents were as follows: 2652 ± 130.93 g for PVOH+BDO, 2459 ± 121.01 g for PVOH+BDO+TP, 2413 ± 119.94 g for PVOH+PDO, and 2293 ± 85.50 g for PVOH+PDO+TP. The control samples and glycerol-containing samples recorded hardness values under 2000 g: 1735 ± 12.22 g for PVOH, 1263 ± 62.17 g for PVOH+TP, 1934 ± 90.30 g for PVOH+Gly, and 1462 ± 52.54 g for PVOH+Gly+TP.

Deformation at hardness indicates the distance values (mm) at hardness point, and for our samples it appeared that biofilms containing glycerol as plasticizer recorded the highest values: 4.55 ± 0.11 mm for PVOH+Gly and 4.61 ± 0.23 mm for PVOH+Gly+TP, while the rest of the samples were beneath these values: 1.23 ± 0.01 mm for PVOH, 1.08 ± 0.05 mm for PVOH+TP, 3.43 ± 0.17 mm for PVOH+BDO, 4.47 ± 0.21 mm for PVOH+BDO+TP, 3.05 ± 0.15 mm for PVOH+PDO, and 3.52 ± 0.17 mm for PVOH+PDO+TP samples.

The hardness work gives the values for the stress that is necessary to overcome the internal strengths of bonds within a specific matrix. In our experiment, biofilms containing BDO recorded the highest values considering the hardness work done, achieving 60.94 ± 3.00 mJ for PVOH+BDO and 58.80 ± 2.76 mJ for PVOH+BDO+TP. The samples containing glycerol recorded 45.28 ± 2.19 mJ (PVOH+Gly) and 37.05 ± 0.18 mJ (PVOH+Gly+TP), while the samples containing PDO recorded 56.33 ± 2.74 mJ (PVOH+PDO) and 34.46 ± 1.17 mJ (PVOH+PDO+TP). The control samples showed the lowest values for the hardness work: 8.85 ± 0.05 mJ (PVOH) and 4.85 ± 0.19 mJ (PVOH+TP).

As hardness is one of the most important properties of a material, allowing it to resist physical deformation [83], it seems that addition of polyols significantly impacted the mechanical behavior of the PVOH-based biofilms. The presence of BDO especially, but also of PDO and glycerol in biofilms created strong bonds within the PVOH matrix by increasing their resistance. On the other hand, the presence of TP also influenced the biofilms’ response to external mechanical stress. Except for the deformation at hardness (mm) where the results increased with the addition of TP, the results registered for hardness (g) and hardness work (mJ) decreased with the addition of TP. The purpose of TP addition to the formulated biofilms was, however, to imprint color on the solid matrix.

## 4. Conclusions

In the present paper, the integration of TP in PVOH to form natural colored biofilms, with the addition of biomass-derived polyols as plasticizer agents for higher flexibility has been reported. Polyols like glycerol, PDO, and BDO positively influenced the physical properties of the PVOH-based biofilms when compared to the control (PVOH). The solid PVOH-based biofilms appeared as flexible as the plastic materials that are commercially available for packaging. From all tested biofilms, the samples that contained glycerol were the most malleable and the softest to touch, followed by samples containing PDO and BDO. Considering the hardness parameter, the highest values were recorded for the biofilms containing BDO and PDO as plasticizers with and without the presence of coloring agents, suggesting that the presence of BDO and PDO, and glycerol in biofilms created strong bonds within the PVOH matrix by increasing their resistance.

The incorporation of Gly, PDO, and BDO in the biofilms provided better thermoplastic processability than their counterparts, PVOH and PVOH+TP. Workability and rigidity issues can be solved with the addition of plasticizers, like glycerol, which enhances the ductility and flexibility of biofilms, while PDO and BDO are able to improve the crystallinity and the thermal characteristics of biofilms.

The natural pigments extracted from TP did not influence the biofilms’ malleability but confirmed their aesthetic impact in coloring the biofilm matrices regardless of the presence of polyols. Biofilms with TP had lower transparency values compared with control biofilms (without vegetal pigments).

Considering their “green” nature and eco-sustainability, the use of naturally-occurring pigments, polymers, and biomass-derived polyols is an innovative, economically-attractive integrated approach for the biodegradable packaging industry.

## Figures and Tables

**Table 1:** Identification and quantification of carotenoids in TP.

Peak No.	Compound	Rt (min)	λmax (nm)	Q (mg/100 DW)
1	Lutein	6.41	448, 474	1.498 ± 0.06
2	Lycopene	13.42	446, 473	0.135 ± 0.01
3	β-carotene	14.51	455, 480	1.605 ± 0.05

**Table 2:** The equilibrium moisture content (%) of PVOH-based formulations. The reported data are the mean values of three independent replicates with their standard deviations (±).

PVOH Formulations	Moisture Content (%)
PVOH	3.75 ± 0.00
PVOH+Gly	26.91 ± 0.04
PVOH+PDO	5.59 ± 0.01
PVOH+BDO	8.04 ± 0.03
PVOH+TP	3.73 ± 0.00
PVOH+Gly+TP	11.12 ± 0.01
PVOH+PDO+TP	4.76 ± 0.01
PVOH+BDO+TP	6.44 ± 0.02

**Table 3:** The transparency of PVOH-based biofilms plasticized with polyols and incorporated with TP.

Polymeric Films	Transparency (nm/mm)
PVOH	0.13 ± 0.02 e
PVOH+Gly	1.10 ± 0.04 c,d
PVOH+PDO	0.76 ± 0.04 d,e
PVOH+BDO	1.62 ± 0.05 c
PVOH+TP	0.16 ± 0.01 e
PVOH+Gly+TP	2.61 ± 0.08 b
PVOH+PDO+TP	3.70 ± 0.11 a
PVOH+BDO+TP	4.05 ± 0.23 a

Values (expressed as mean values ± SD, n = 3) in the same column followed by different letters (a–g) indicate significant differences (*p* < 0.05) between types of polymers. (One-way ANOVA–Tukey’s multiple comparison test (*p* = 0.05), GraphPad Prism Version 8.0.1, Graph Pad Software, Inc., San Diego, CA, USA).

## Supplementary Materials

The following are available online at https://www.mdpi.com/2073-4360/12/3/532/s1. Table S1: Shear viscosity and shear stress of biofilms at 37 °C temperature with standard deviation. Table S2: Shear viscosity and shear stress of biofilms at 30 °C temperature with standard deviation. Table S3: Shear viscosity and shear stress of biofilms at 21 °C temperature with standard deviation.
