# Supplementary material for: Poly(vinyl alcohol)-Based Biofilms Plasticized with Polyols and Colored with Pigments Extracted from Tomato By-Products
Source: Polymers (Basel). 2020 Mar 2;12(3):532. doi: 10.3390/polym12030532 (PMC7182853; doi:10.3390/polym12030532)
Supplement: Supplementary file 1 [file polymers-12-00532-s001.pdf]

Supplementary material 1. Shear viscosity and shear stress of biofilms at 37 °C with standard deviation (±).

37 °C	S.R. [1/s]	5	8	12	19	29	46	71	111	173	269
**PVOH**	*Visc.* [mPa·s]	1.18± 0.09	4.70± 0.04	7.90± 0.08	7.06± 0.05	9.01± 0.09	9.06± 0.10	8.81± 0.12	8.85± 0.07	8.9± 0.08	8.24± 0.09
	S.S. [Pa]	0.006± 0.005	0.036± 0.02	0.08± 0.03	0.133± 0.04	0.263± 0.02	0.373± 0.01	0.596± 0.02	0.938± 0.02	1.425± 0.05	2.181± 0.02
**PVOH + TP**	*Visc.* [mPa·s]	N.D.	5.31± 0.02	5.62± 0.09	7.89± 0.11	9.005± 0.16	9.06± 0.10	8.81± 0.07	8.85± 0.09	8.90± 0.05	9.00± 0.10
	S.S. [Pa]	N.D.	0.041± 0.05	0.07± 0.02	0.148± 0.03	0.272± 0.05	0.414± 0.06	0.631± 0.08	0.982± 0.07	1.565± 0.10	2.371± 0.09
**PVOH + Gly**	*Visc.* [mPa·s]	5.32± 0.07	9.58± 0.02	11.46± 0.11	14.85± 0.16	14.55± 0.09	14.65± 0.02	14.13± 0.06	13.97± 0.08	13.99± 0.08	13.99± 0.06
	S.S. [Pa]	0.011± 0.006	0.075± 0.07	0.143± 0.08	0.283± 0.10	0.422± 0.09	0.651± 0.10	1.005± 0.09	1.546± 0.11	2.424± 0.12	3.755± 0.08
**PVOH + Gly + TP**	*Visc.* [mPa·s]	5.27± 0.07	9.41± 0.09	12.02± 0.08	14.57± 0.11	14.56± 0.10	14.24± 0.14	14.12± 0.07	13.96± 0.08	14.04± 0.12	13.97± 0.08
	S.S. [Pa]	0.013± 0.005	0.066± 0.06	0.155± 0.09	0.301± 0.11	0.452± 0.05	0.598± 0.10	1.012± 0.11	1.421± 0.12	2.356± 0.13	3.825± 0.09
**PVOH + PDO**	*Visc.* [mPa·s]	9.89± 0.12	7.89± 0.10	10.16± 0.12	13.99± 0.05	13.75± 0.06	13.87± 0.09	13.79± 0.04	13.99± 0.08	14.01± 0.10	13.98± 0.08
	S.S. [Pa]	0.015± 0.009	0.081± 0.07	0.201± 0.11	0.321± 0.07	0.398± 0.07	0.605± 0.11	1.099± 0.10	1.564± 0.13	2.365± 0.13	3.831± 0.05
**PVOH + PDO + TP**	*Visc.* [mPa·s]	10.02± 0.11	8.56± 0.11	10.22± 0.09	14.02± 0.08	13.95± 0.03	14.05± 0.05	13.98± 0.07	14.01± 0.11	13.99± 0.08	14.03± 0.12
	S.S. [Pa]	0.009± 0.002	0.099± 0.04	0.197± 0.10	0.431± 0.08	0.402± 0.08	0.681± 0.12	1.102± 0.11	1.632± 0.14	2.402± 0.14	3.956± 0.09
**PVOH + BDO**	*Visc.* [mPa·s]	12.35± 0.09	9.87± 0.11	10.93± 0.07	13.87± 0.08	13.89± 0.11	13.98± 0.09	14.05± 0.11	13.89± 0.08	13.99± 0.13	14.32± 0.07
	S.S. [Pa]	0.035± 0.005	0.114± 0.08	0.281± 0.11	0.298± 0.06	0.356± 0.05	0.589± 0.14	1.159± 0.20	1.602± 0.09	2.412± 0.10	3.980± 0.07
**PVOH + BDO+ TP**	*Visc.* [mPa·s]	13.55± 0.11	10.88± 0.13	11.02± 0.09	14.02± 0.08	14.03± 0.09	13.99± 0.01	14.03± 0.03	14.09± 0.09	14.12± 0.15	14.68± 0.09
	S.S. [Pa]	0.041± 0.006	0.123± 0.09	0.320± 0.10	0.356± 0.077	0.409± 0.07	0.603± 0.12	1.162± 0.08	1.599± 0.15	2.416± 0.11	3.956± 0.09

Visc. – viscosity, *S.S.* – shear stress, *S.R.* – shear rate

Supplementary material 2. Shear viscosity and shear stress of biofilms at 30 °C with standard deviation (±).

**30 °C**	**S.R. [1/s]**	**5**	**8**	**12**	**19**	**29**	**46**	**71**	**111**	**173**	**269**
**PVOH**	*Visc.* [mPa·s]	0.47± 0.05	4.23± 0.12	6.52± 0.14	9.25± 0.13	9.34± 0.07	10.07± 0.09	9.79± 0.11	9.74± 0.13	9.68± 0.10	9.67± 0.15
	S.S. [Pa]	0.003± 0.001	0.027± 0.03	0.079± 0.05	0.173± 0.06	0.278± 0.04	0.457± 0.03	0.702± 0.07	1.079± 0.10	1.658± 0.07	2.567± 0.04
**PVOH + TP**	*Visc.* [mPa·s]	8.81± 0.11	9.75± 0.06	10.23± 0.14	10.26± 0.13	11.39± 0.08	11.04± 0.05	11.14± 0.11	10.92± 0.18	10.86± 0.07	10.80± 0.14
	S.S. [Pa]	0.044± 0.02	0.076± 0.009	0.126± 0.06	0.196± 0.08	0.337± 0.06	0.501± 0.03	0.784± 0.04	1.201± 0.06	1.869± 0.04	2.893± 0.11
**PVOH + Gly**	*Visc.* [mPa·s]	6.36± 0.09	6.62± 0.12	8.91± 0.08	9.74± 0.12	10.94± 0.05	10.87± 0.03	10.66± 0.09	10.62± 0.11	10.55± 0.08	10.56± 0.12
	S.S. [Pa]	0.031± 0.01	0.052± 0.08	0.126± 0.02	0.206± 0.11	0.351± 0.05	0.546± 0.13	0.837± 0.03	1.309± 0.10	2.033± 0.09	3.108± 0.05
**PVOH + Gly + TP**	*Visc.* [mPa·s]	6.40± 0.11	6.69± 0.14	9.04± 0.09	9.97± 0.19	11.09± 0.13	10.71± 0.07	10.56± 0.05	10.59± 0.12	10.58± 0.09	10.37± 0.08
	S.S. [Pa]	0.032± 0.009	0.052± 0.02	0.101± 0.05	0.188± 0.09	0.326± 0.10	0.489± 0.11	0.752± 0.15	1.174± 0.09	1.827± 0.11	2.785± 0.07
**PVOH + PDO**	*Visc.* [mPa·s]	7.49± 0.10	10.11± 0.09	7.81± 0.07	8.79± 0.12	10.93± 0.11	10.82± 0.13	10.87± 0.07	10.72± 0.14	10.85± 0.09	10.78± 0.06
	S.S. [Pa]	0.041± 0.01	0.084± 0.05	0.121± 0.10	0.196± 0.09	0.369± 0.11	0.554± 0.05	0.857± 0.08	1.321± 0.10	2.065± 0.11	3.191± 0.08
**PVOH + PDO + TP**	*Visc.* [mPa·s]	7.52± 0.13	10.06± 0.13	7.76± 0.03	8.82± 0.14	10.86± 0.06	10.78± 0.13	10.87± 0.08	10.68± 0.09	10.74± 0.11	10.65± 0.07
	S.S. [Pa]	0.038± 0.008	0.078± 0.09	0.094± 0.04	0.166± 0.10	0.319± 0.11	0.493± 0.07	0.773± 0.06	1.183± 0.12	1.185± 0.17	2.862± 0.08
**PVOH + BDO**	*Visc.* [mPa·s]	4.11± 0.04	6.17± 0.13	8.85± 0.11	9.99± 0.06	10.73± 0.10	11.29± 0.06	10.89± 0.17	10.81± 0.06	10.69± 0.10	10.59± 0.08
	S.S. [Pa]	0.026± 0.007	0.064± 0.09	0.128± 0.10	0.243± 0.07	0.378± 0.08	0.553± 0.11	0.876± 0.14	1.343± 0.10	2.069± 0.08	3.178± 0.12
**PVOH + BDO+ TP**	*Visc.* [mPa·s]	4.03± 0.06	6.22± 0.12	8.94± 0.05	9.93± 0.05	10.61± 0.14	11.34± 0.09	10.94± 0.07	10.89± 0.08	10.77± 0.09	10.64± 0.12
	S.S. [Pa]	0.021± 0.009	0.048± 0.02	0.108± 0.08	0.187± 0.18	0.312± 0.05	0.518± 0.17	0.778± 0.08	1.207± 0.15	1.858± 0.14	2.857± 0.07

Visc. – viscosity, *S.S.* – shear stress, *S.R.* – shear rate

Supplementary material 3. Shear viscosity and shear stress of biofilms at 21 °C with standard deviation (±).

21 °C	S.R. [1/s]	5	8	12	19	29	46	71	111	173	269
**PVOH**	*Visc.* [mPa·s]	5.84± 0.07	7.35± 0.05	11.98± 0.09	13.04± 0.10	12.82± 0.05	13.27± 0.12	13.09± 0.08	13.03± 0.11	13.11± 0.08	12.94± 0.09
	S.S. [Pa]	0.029± 0.009	0.057± 0.05	0.139± 0.06	0.244± 0.07	0.368± 0.06	0.595± 0.04	0.918± 0.04	1.433± 0.03	2.224± 0.08	3.428± 0.07
**PVOH + TP**	*Visc.* [mPa·s]	7.56± 0.07	9.25± 0.07	10.56± 0.11	13.56± 0.13	14.45± 0.12	15.01± 0.09	15.12± 0.07	14.95± 0.08	14.87± 0.06	14.84± 0.12
	S.S. [Pa]	0.043± 0.008	0.075± 0.06	0.135± 0.05	0.27± 0.07	0.437± 0.04	0.686± 0.11	1.047± 0.08	1.621± 0.09	2.506± 0.11	3.912± 0.07
**PVOH + Gly**	*Visc.* [mPa·s]	1.31± 0.08	4.98± 0.11	6.84± 0.13	8.56± 0.11	8.64± 0.08	8.79± 0.09	8.76± 0.07	8.65± 0.09	8.60± 0.05	8.57± 0.11
	S.S. [Pa]	0.017± 0.008	0.056± 0.09	0.103± 0.11	0.202± 0.14	0.291± 0.10	0.444± 0.08	0.693± 0.17	1.081± 0.13	1.665± 0.05	2.593± 0.08
**PVOH + Gly + TP**	*Visc.* [mPa·s]	0.84± 0.09	5.22± 0.07	6.66± 0.07	8.61± 0.13	8.69± 0.12	8.54± 0.17	8.39± 0.07	8.68± 0.11	8.51± 0.04	8.49± 0.06
	S.S. [Pa]	0.011± 0.001	0.039± 0.04	0.079± 0.11	0.155± 0.15	0.248± 0.08	0.401± 0.13	0.621± 0.12	0.97± 0.17	1.495± 0.03	2.324± 0.05
**PVOH + PDO**	*Visc.* [mPa·s]	1.02± 0.14	2.48± 0.12	5.81± 0.11	7.34± 0.07	8.81± 0.10	9.33± 0.11	9.17± 0.05	9.22± 0.09	9.15± 0.08	9.07± 0.05
	S.S. [Pa]	0.041± 0.01	0.083± 0.08	0.121± 0.13	0.196± 0.15	0.369± 0.09	0.554± 0.10	0.857± 0.07	1.32± 0.06	2.064± 0.14	3.191± 0.04
**PVOH + PDO + TP**	*Visc.* [mPa·s]	1.09± 0.10	2.36± 0.13	6.02± 0.04	7.45± 0.07	8.77± 0.08	9.21± 0.11	9.19± 0.07	9.30± 0.13	9.25± 0.07	9.14± 0.11
	S.S. [Pa]	0.038± 0.007	0.078± 0.05	0.094± 0.11	0.166± 0.09	0.318± 0.07	0.493± 0.11	0.773± 0.12	1.183± 0.17	1.852± 0.08	2.861± 0.10
**PVOH + BDO**	*Visc.* [mPa·s]	1.52± 0.07	5.21± 0.14	7.94± 0.08	8.25± 0.07	8.98± 0.12	8.76± 0.04	8.65± 0.12	8.69± 0.09	8.54± 0.11	8.37± 0.05
	S.S. [Pa]	0.026± 0.009	0.064± 0.07	0.128± 0.13	0.243± 0.07	0.378± 0.09	0.553± 0.11	0.876± 0.11	1.343± 0.15	2.069± 0.07	3.178± 0.09
**PVOH + BDO+ TP**	*Visc.* [mPa·s]	1.75± 0.14	5.41± 0.11	8.02± 0.07	8.34± 0.09	8.78± 0.10	8.81± 0.03	8.75± 0.06	8.73± 0.07	8.68± 0.14	8.71± 0.08
	S.S. [Pa]	0.007± 0.001	0.038± 0.07	0.102± 0.11	0.159± 0.08	0.273± 0.09	0.389± 0.13	1.615± 0.11	0.937± 0.10	1.461± 0.07	2.251± 0.11

Visc. – viscosity, *S.S.* – shear stress, *S.R.* – shear rate
